# Alveolar type 2 progenitor cells for lung injury repair

**DOI:** 10.1038/s41420-019-0147-9

**Published:** 2019-02-08

**Authors:** Ayobami Matthew Olajuyin, Xiaoju Zhang, Hong-Long Ji

**Affiliations:** 1 Department of Respiratory Medicine, Henan Provincial People’s Hospital, Henan University, Zhengzhou Henan, China; 2 Department of Cellular and Molecular Biology, University of Texas Health Science Center at Tyler, Tyler, TX USA; 3 Texas Lung Injury Institute, University of Texas Health Science Center at Tyler, Tyler, TX USA

## Abstract

Alveolar type 2 progenitor cells (AT2) seem closest to clinical translation, specifying the evidence that AT2 may satisfactorily control the immune response to decrease lung injury by stabilizing host immune-competence and a classic and crucial resource for lung regeneration and repair. AT2 establish potential in benefiting injured lungs. However, significant discrepancies linger in our understanding vis-à-vis the mechanisms for AT2 as a regime for stem cell therapy as well as essential guiding information for clinical trials, including effectiveness in appropriate pre-clinical models, safety, mostly specifications for divergent lung injury patients. These important gaps shall be systematically investigated prior to the vast therapeutic perspective of AT2 cells for pulmonary diseases can be considered. This review focused on AT2 cells homeostasis, pathophysiological changes in the pathogenesis of lung injury, physiological function of AT2 cells, apoptosis of AT2 cells in lung diseases, the role of AT2 cells in repairing processes after lung injury, mechanism of AT2 cells activation promote repairing processes after lung injury, and potential therapy of lung disease by utilizing the AT2 progenitor cells. The advancement remains to causally connect the molecular and cellular alteration of AT2 cells to lung injury and repair. Conclusively, it is identified that AT2 cells can convert into AT1 cells; but, the comprehensive cellular mechanisms involved in this transition are unrevealed. Further investigation is mandatory to determine new strategies to prevent lung injury.

## Facts

- Due to the fact that the production of surfactant in rodent and human are unsimilar, hence investigations on protein synthesis, phospholipid synthesis and assembly in human AT2 cells are interesting for further studies.
- Apoptosis of AT2 cell is associated with the pathogenesis of lung injury
- It is promising that sustaining Notch signaling might reduce effective lung repair by extending inflammation, as well as by regulating progenitor identity, while this remains to be exploited
- New approaches to treat lung injury can be further unraveled by using AT 2 progenitor cells

## Open questions

- The precise mechanism of AT2 apoptosis in ALI/ARDS, COPD and IPF is still debatable
- Whether the increased PAI‐1 expression is liable for AT2 cell senescence in fibrotic lung diseases and, most essentially, how PAI‐1 promotes cell senescence remain indistinguishable
- Distinguishing whether the transporter ABCA3 is essential for lamellar body biogenesis and similarly regulation of phospholipid import and specificity
- The causes controlling baseline of alveolar fluid volume and pH remain unclear.
- The importance of the sodium-phosphate transporter situated on the apical membrane of AT2 cells and exactly how the other components of alveolar fluid are processed are limited.
- Investigating the significance of EMT and epigenetics to pulmonary fibrosis will be a fascinating study.
- It is also interesting to investigate the effects of ROS (hydrogen peroxides, nitric oxide, and hydroxide) on induced DNA damage and repair through the differentiation of AT 2 progenitor cells.
- The significance of mitochondrial complexes I and III, NADPH oxidase isoform NOX4 during AT2 cell differentiation and mechanisms underlying the processes will be fascinating to study.

## Introduction

Acute lung injury (ALI) and acute respiratory distress syndrome (ARDS) are the major cause of death in critical care, with a mortality rate of around 40%. In the US only, there are 200,000 new cases per annum^1^. ALI/ARDS also form a significant lasting illness and public health problem, with major neuromuscular, respiratory and mental dysfunction found in 50–70% of survivors, and just 49% able to work one-year post-discharge^2^. Notwithstanding being a focus of current rigorous research determinations over four decades, there are no effective specific except supportive interventions for ALI/ARDS^3^. Extensive clinical trials of several therapeutic strategies are all failed, including nitric oxide, anti-oxidants^4^, surfactants^5^, corticosteroids^6^, immunomodulating agents^4^, and granulocyte-macrophage-colony-stimulating factor^7^.

To date, improvement in the management of ALI/ARDS rarely relies on general supportive measures, e.g., preventive mechanical ventilation^3^, regulative intravenous fluid management^8^, and prone position of seriously hypoxaemic patients^9^. While these maneuvers have decreased mortality in ICU patients^10^, the disappointment of pharmacologic therapies proposes the necessity to contemplate novel methods for ALI/ARDS. ALI/ARDS is exceedingly heterologously pathogenic diseases with multiple phenotypes. Previous concepts of distinct disease phases, from an early ‘proinflammatory’ to a later ‘fibrotic’ phase, now seem to be an over-simplification. These ‘phase’ abundantly exist, with the denotation of pro-inflammatory effect resulting to host injury. In the ALI/ARDS, there is the presence of an incapacitated immune response to pathogens, regeneration, and fibrosis. Hence, the different strategies used for therapeutics have been unsuccessful.

**Fig. 1:**
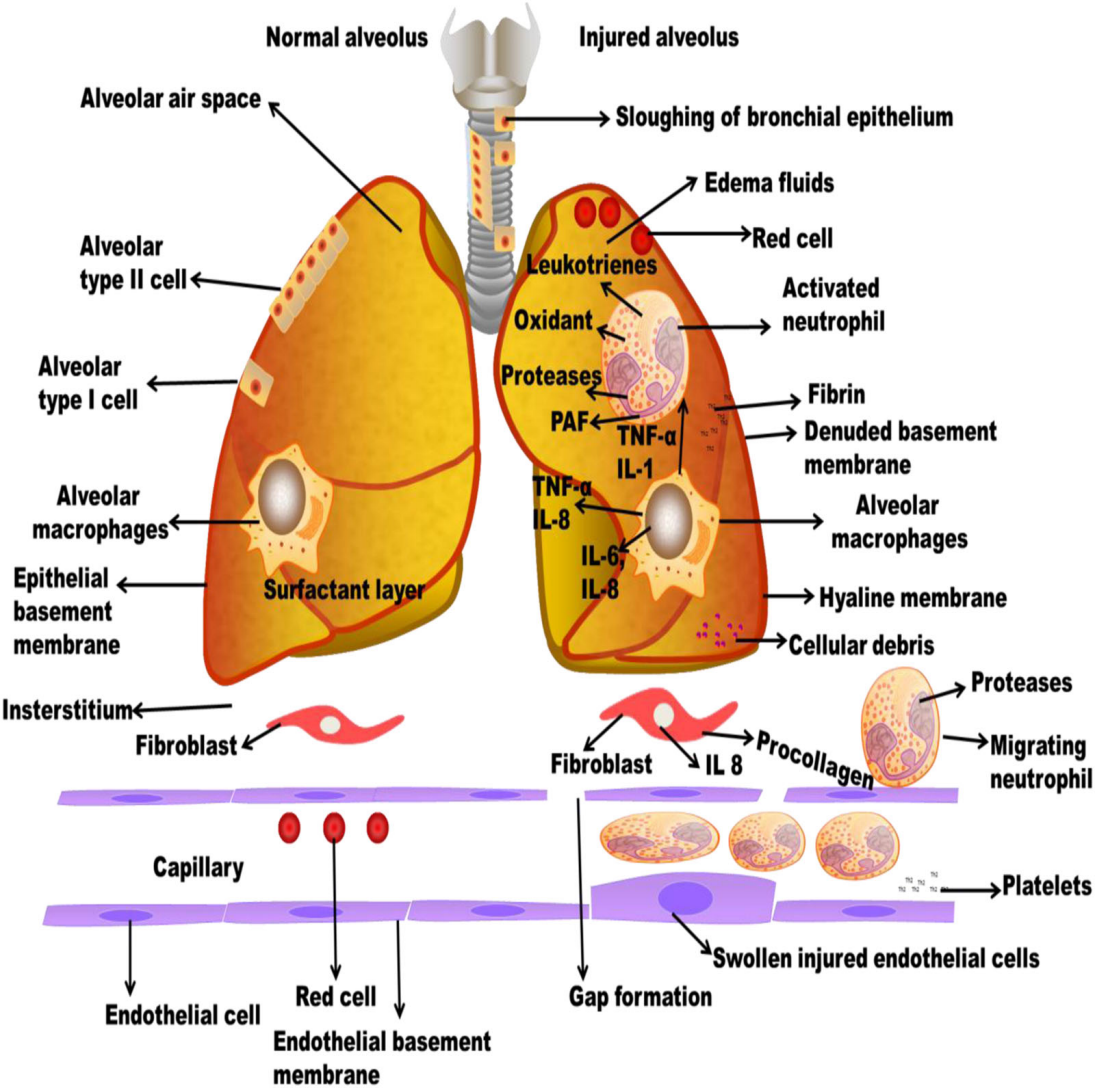
**Pathophysiology of lung injury**

Generally, many of the lung injury diseases are related to aging^11^ (Fig. 1). Chronic obstructive pulmonary disease (COPD) has elevated to become the fourth prominent reason for morbidity globally. There is an emergent discovery that aging is associated with the pathogenesis of a number of chronic lung diseases; really, most lung diseases are either mostly limited to the elderly. The occurrence of COPD was likely at 3.2% among those aged 25–44 years and 10.3% among those 65–74 years in the United States^12^. Likewise, the death related to COPD and pneumonia^13^ and the occurrence of idiopathic pulmonary fibrosis (IPF) all nurture with aging and has been linked with elevated vulnerability to both viral and bacterial pneumonia. Development of ARDS is more visible in older patients. The major risk factors for ARDS are pneumonia and sepsis which occur majorly in old patients^14^.

Lung has the AT2 progenitor cells which are involving in the crucial role in ALI/ARDS repair. The mature lung comprises four main biologically different portions including, the trachea, bronchi, bronchioles, and alveoli, and respectively has a specific stem/progenitor population^15^. Alveolar type 1 (AT1) and alveolar type 2 (AT2) cells are majorly found in the gaseous alveolar surfaces^16^. AT1 cells are more sensitive to injuries than AT2 cells^17^.

Once AT1 cells are injured, adjacent AT2 cells are stimulated to multiply and transdifferentiate into AT1 cells. Consequently, in the alveoli the AT2 cells have long been thought to function as progenitor cells^18^. Current studies on rodent models have recognized stem/progenitor populations for alveolar epithelial cells, and have discovered that the stem/progenitor populations have an essential function in lung repair and tumorigenesis^19^. The healthy human lung comprises of the cuboidal AT2 pneumocyte (15%) of total cells^20^. The surfactant protein (SP) C-expressing embryologic precursor is majorly found in AT2 cell^21^. In most small-animal species (with the remarkable exclusion of the morphologically advanced guinea pig^22^, alveolarization occurs after birth. More investigation is needed in the mechanisms regulating the transition of primitive saccules to mature alveoli.

AT2 cells cycle every 28–35 days in the adult rodent lung, and this slow mitotic rate is also presumed to occur in humans^23^. This rate of biochemical reaction is improved in response to lung injury^24^ and growth factors such as keratinocyte growth factor (KGF)^23^. Investigations of adult AT2 cells arising from cells other than AT2 cells themselves were conducted in the last few years. These include SPC, Clara cell secretory protein (CCSP) cells at the bronchoalveolar junction (bronchoalveolar stem cells)^25^, and extensive diversity of cells derived from the circulation^26^. Furthermore, an analogous population is still to be discovered in humans. The investigation including cre-lox lineage tracing in mice shows only a slight involvement of bone marrow to the AT2 cell pool^27^. This type of nuclear rearrangement leads to morphologically and karyotypically aberrant cells that seem to have come through cell–cell fusion^28^. The biological importance of this finding in the healthy lung is viewed as minimal, given current data. The contribution of AT2 phenotype and bone marrow cells to the pathogenesis of lung injury is still debatable. This indicates the necessity to reckon unique therapeutic strategy at minimizing early injury while protective host immune capability and improving lung regeneration and repair. Hence, it is a hot topic to answer the question of whether alveolar epithelial progenitor stem cells-AT2 could fit this new therapeutic model. We herein briefly summarized the key progress of this field and discuss future directions.

### Pathophysiological alteration of lung injury

**Fig. 2:**
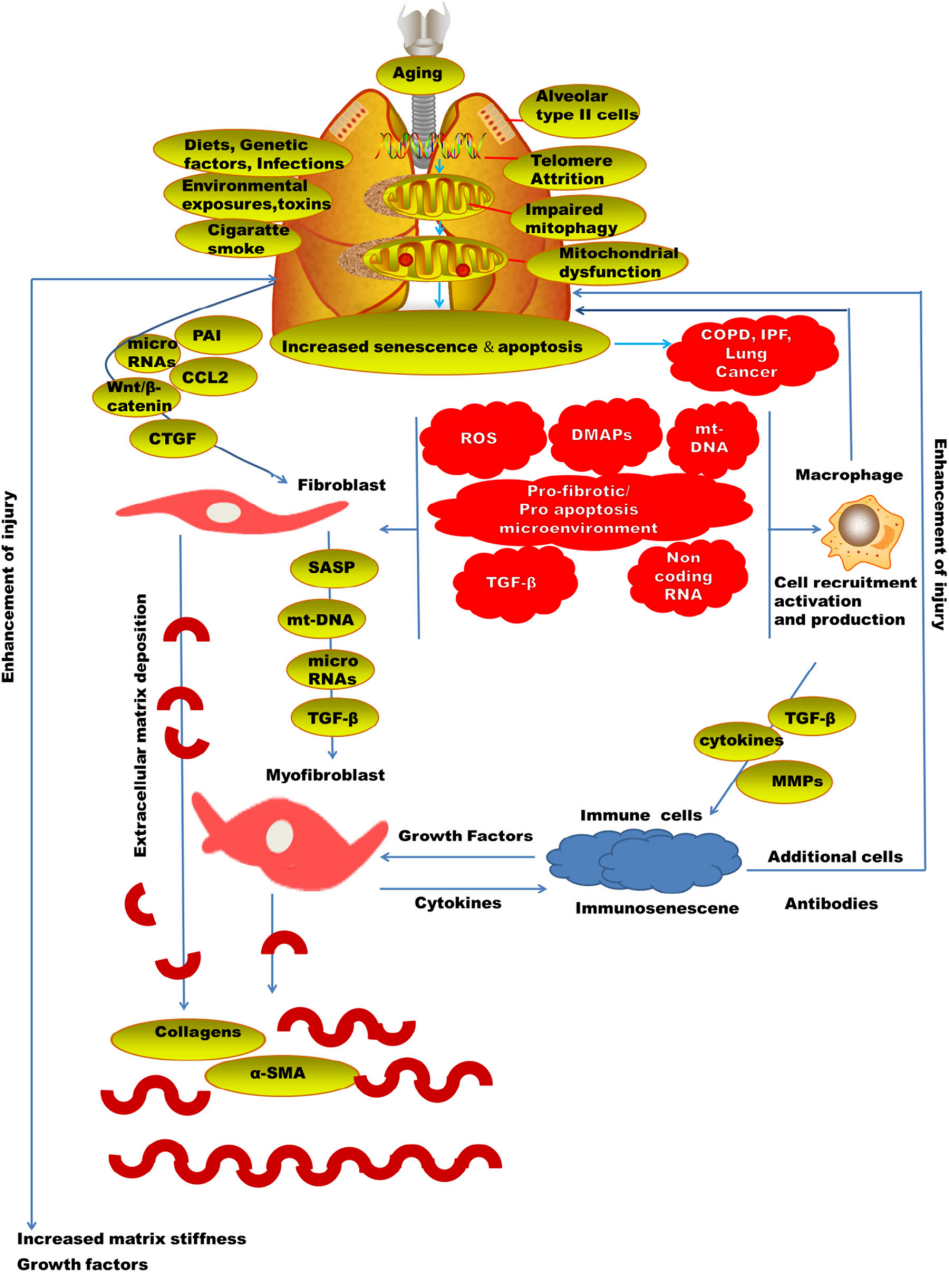
**Pathophysiology of COPD and IPF**

ALI is also named as chronic inflammation which found in the alveolar-capillary membrane. The molecular constituents such as microvascular endothelium, alveolar epithelium, and specialized fibroblasts occur at the initial phase. The magnitude of alveolar epithelial damage is the significant prognosticator of consequence^29^ (Fig. 2). Distinctive pathohistological appearances contain widespread necrosis of AT1 cells and the presence of protein-rich hyaline membranes on an uncovered basement membrane. Loss of alveolar epithelial reliability leads to the accumulation of protein-rich and exceedingly cellular edema fluid in the interstitium and alveoli. This inflammatory milieu contains mainly of activated neutrophils and alveolar macrophages, which secrete inflammatory mediators that disorder epithelial fluid transport and impaired surfactant production of AT2 cells. Capillary thrombosis and extravascular fibrin accumulation formed as an effect of endothelial-dysfunction-associated upregulation and activation of tissue factor, and damage of the ability to activate the vitamin-K-dependent proteins C and S. This local pro-coagulant state potentiate pulmonary dysfunction and the acute inflammatory response^30^. VALI aggravates the syndrome more by physically disorderly responsible tissues and cells, leading to the expression of pro-inflammatory and/or pro-fibrotic mediators^31^. Lung injury is distributed by pro-inflammatory and systemic inflammation, multiple-organ dysfunction, and apoptosis connected with VALI.

Subsequent to the acute inflammatory phase, most patients steadily convalesce normal lung function. Epithelial growth factor improves the multiplication of AT2 cells, which are also understood to act as progenitor cells for both daughter AT2 cells and AT1 cells^32^. This phenomenon is combined to reconstruct the epithelial barrier and reinstate normal lung function.

Cuboidal AT2 cells are more impervious to injury^33^. Surfactant production and ion transport functions are present in AT2 cells. They also function as progenitor cells for renewal of AT1 cells subsequent to injury. Alveolar epithelial damages in the previous ultrastructural investigation of patients dying with ALI/ARDS include a spectrum from cytoplasmic swelling, vacuolization, and bleb formation to necrosis and widespread denuding of epithelial cells^34^. Injury to AT2 cells also prevents surfactant synthesis and turnover, leading to the irregularities of both the lipid and protein constituents of surfactant that are features of ALI/ARDS^35^. Increased permeability pulmonary edema promotes impairment of the surfactant because of manifestation of serum proteins^36^ and proteolytic enzymes^37^ in the alveolar space.

AT2 cells can self‐renew and also differentiate into AT1 cells and so are referred to as alveolar progenitor cells^38^. AT2 cell senescence is evident in IPF^39^ and in experimental fibrosis models^40^. A recent disease model is that lung fibrosis grows as a result of constant insults plus genetic and senescence-related hazard factors, resulting in alveolar epithelial cell impairment, which is followed by activation of myofibroblasts and replacement of injured alveolar epithelium with fibrotic tissue, due to a reduced reparative capability of alveolar epithelium. Clarification of the mechanisms underlying AT2 cell senescence, consequently, may be a considered approach to the understanding of the disease pathogenesis and thus the exploitation of efficient therapeutics.

Plasminogen is converted into plasmin by serpine, a serine proteinase playing a key role in fibrinolysis^41^. Besides suppression of fibrinolysis, PAI‐1 has numerous other roles, including modulation of cell adhesion, relocation, and multiplying, unautonomous or autonomous of its protease inhibitory activity^42^. Investigations from others have displayed that PAI‐1 shows a critical role in the progress of lung fibrosis, while the mechanism whereby PAI‐1 stimulates lung fibrosis remains unclear. Significantly, PAI‐1 expression is enlarged in senescent cells^43^ and developing confirmation proposes that PAI‐1 is not only a marker nevertheless a facilitator of cell senescence. Nevertheless, whether the increased PAI‐1 expression is liable for AT2 cell senescence in fibrotic lung diseases and, most essentially, how PAI‐1 promotes cell senescence remain indistinguishable.

The gradual degeneration in the functional ability of an organism predisposing to death is aging^44^. IPF is a disease which is associated with aging^45^, with accumulative occurrence and predominance in human subjects over the age of 50 years^46^. There is a high rate of death in individual diagnosed of IPF^47^. Patient diagnosed of progeria syndromes are also at a higher possibility of fibrotic disorder^48^. Telomere dysfunction syndromes and the short telomere length is a dangerous factor in an individual with IPF^49^.

Previously, infiltrating leukocytes were understood to be significant to the pathology while the epithelium was thought to target the injury. However, current investigation reveals that the epithelium is a rich source of molecules involved in modulating inflammation and lung defense mechanisms. The human AT2 epithelial cell expression of TLR-4 was investigated by previous researchers^50^ and revealed a comprehensive spectrum of cytokines and chemokines including IL-1β, TNF-α, IL-6, CCL2, CXCL8, CXCL1, and CCL20 (MIP-3α) after treatment with LPS^51^. When they are exposed to LPS, AT2 cells produce more chemokines than alveolar macrophages from the same subject. In TLR4 signaling mechanism, the AT2 cell serves as a major source of neutrophil chemoattractant chemokines^52^.

### The physiological function of AT2 cells

AT2 cells are important for the production of surfactant. The AT2 cell secretes, synthesizes, and reutilizes the protein and lipid constituents of pulmonary surfactant. The unique part of importance is the synthesis of dipalmitoylphosphatidylcholine and the function of phosphatidylcholine remodeling through a highly specific deacylation/reacylation reaction^53^. Due to the fact that the production of surfactant in rodent and human are unsimilar, hence investigations on protein and phospholipid synthesis and assembly in human AT2 cells are interesting for further studies. Other questions concern the lamellar body. Distinguishing whether the transporter ABCA3 is essential for lamellar body biogenesis^54^ similarly controls phospholipid import and specificity. Similarly, the authentic role of surfactant proteins in these processes is indistinguishable. The thoughtful respiratory failure caused by the deficiency of SPB^55^ indicates this protein’s significance in surfactant processing and function. The chronic, fibrotic phenotype of SPC deficiency in certain mouse strains and humans suggests a dissimilar, but probably equally significant function for this protein^56^. These problems highlight the need for more evidence in this area. There are no known clinical means of increasing the endogenous pool of surfactant in ALI.

Transepithelial transport in human and rodent AT2 cells assist the alveolar space reasonably free of fluid and transport sodium through well-defined apical sodium channels and the basolateral Na^+^/K^+^-ATPase^57^. Recent in vivo studies using siRNA to knockdown alpha-ENaC (epithelial Na channel) expression found that deletion of this transporter reduced baseline lung fluid absorption by ~30%^58^. However, in ALI, the fluid has a high protein concentration and the epithelial barrier is not intact. The causes controlling baseline of alveolar fluid volume and pH remain unclear. Hence, the importance of the sodium phosphate transporter situated on the apical membrane of AT2 cells^59^ and exactly how the other components of alveolar fluid are processed are limited.

In answer to a diversity of AT1 cell injuries, hyperplastic AT2 cells^60^ conceal the basement membrane and then differentiate into AT1 cells, maintain their AT2 cell phenotype, or undergo apoptosis^61^. The causes regulating induction, differentiation, and clearance of hyperplastic AT2 cells are still unclear. Repopulation of AT2 cell in the normal lung is also unclear. Hence, there are some suggestions that AT2 cells may undergo epithelial to mesenchymal transition (EMT)^62^. Therefore, investigating the significance of EMT and epigenetics to pulmonary fibrosis will be a fascinating study. The importance of the paracrine signaling and molecular mechanism of injured cells remains unexplored. AT2 cells express major histocompatibility class II antigens^63^ but little is known about their ability to present antigen and initiate inflammatory responses and respiratory viruses.

### Apoptosis of AT2 cells in lung diseases

**Fig. 3:**
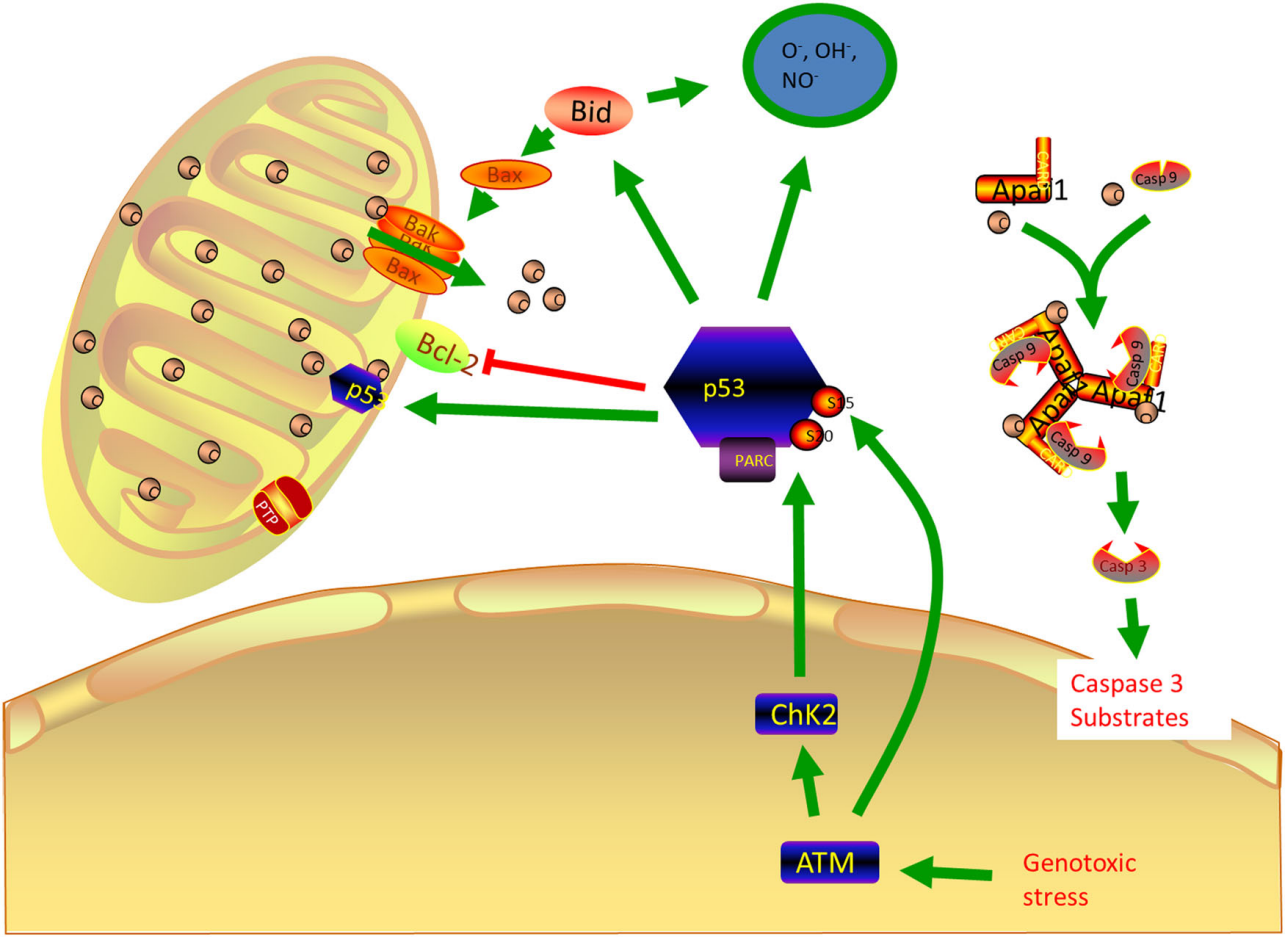
**Roles of p53 in Apoptosis of lung injury**

Apoptosis is a process of programmed cell death. There are majorly two types of apoptosis, which includes intrinsic (mitochondria-mediated) and extrinsic types (receptor-mediated). The intrinsic is also known as apoptosome mediated apoptosis. It is originated majorly in the cytosol. The internal and external stimuli may activate or inhibit the process^64^. When the AT2 cell in ALI/ARDS, COPD, and IPD is injured it will lead to DNA damage which causes protein like ataxia-telangiectasia mutated (ATM), checkpoint kinase 1 (CHK1) which coordinate DNA damage responds, cell cycle arrest which leads to cell death. When they sense the damage, they will also activate the p53 which is a dangerous protein that can turn on multiple proteins. The cell cycle will not be able to proceed to the next stage because of the presence of p53^65^. The major factors controlling the mitochondria-mediated pathways are p53 and bcl2^66^ (Fig. 3). Bcl2 is a mitochondrial outer membrane permeabilization protein which roles are lengthening cellular survival via inhibition of a diversity of apoptotic demises, whether these are p53 dependent or independent^67^. P53 will recruit other protein like p21, BAX (which can create pores in the mitochondria) which allows cytochrome C into the cytosol. When cytochrome C is released, it acts as the death signal. Cytochrome C combines with apoptotic protease activating factor 1 (APAF 1) to activate procaspase 9 to caspase 9 which further activate procaspase 3 to caspase 3. Caspase 3 activates nuclease enzymes which can migrate into the nucleus and degrade the DNA.

Extrinsic types (receptor-mediated) occurs when Fas receptor binds to Fas ligand (FasL) due to infections, DNA damage, and injury. The infections bind to FasL receptor, which in turns activate the death domain receptor and the activation is autocatalytic. Due to the accumulation of death domain signal, it leads to the death-inducing signaling complex, which will activate procaspase 8 to caspase 8. Caspase 8 will activate procaspase 3 to caspase 3, which can degrade the inhibitor of nuclease enzymes and migrate to the nucleus to degrade the DNA. Apoptosis of AT2 cell is assumed to be mainly accountable for the vanishing of surplus epithelial cells during the resolution phase of ALI^68^. The investigation has been done on BALF from ALI/ARDS patients and revealed increased in soluble Fas (Apo1, CD95) and FasL^69^, suggesting that the Fas system might be relevant in the programmed cell death in ALI or ARDS^70^.

Apoptosis of AT2 cell is associated with the pathogenesis of lung fibrosis likewise to its resolution in current investigations^71^. In the fibrotic lung, apoptosis of inflammatory cells might also be advantageous^72^. An authenticity of apoptosis called DNA fragmentation was discovered in bronchiolar cells and AT2 within lung biopsies from patients with IPF and rats with bleomycin-induced lung fibrosis^73^. Thus epithelial apoptosis colocalizes with myofibroblasts where collagen deposition is severe, in patients with IPF. Apoptosis within AT2 cells of fibrotic human lungs was revalidated and discovered of fragmented DNA^74^. Constant with those findings, the “death receptor” Fas was found to be expressed in AT2 within the lungs of IPF patients by numerous researchers^75^. In animal models, similar clarifications were attained^76^. Furthermore, knockout mice lacking the receptor Fas were found to be unaffected to the profibrotic effect of bleomycin. Thus the functions of Fas-induced apoptosis in the development of the pulmonary fibrotic response are still debatable; in addition, pathways other than Fas can initiate epithelial apoptosis and facilitate fibrogenesis. Furthermore, the regulation of apoptosis of AT2 cells may be due to the modifications in the expression of various antiapoptotic and proapoptotic factors (p53 and p21). It has been found out that ERK decreased and active JNK increased in epithelial cells which are important signaling pathway which may elucidate the effects of apoptosis AT2 cells in IPF patients^77^. During the intermediate stage of IPF, TUNEL-positive cells and activated p38 MAPK were found in the in AT2 cells^78^. The main molecular mechanism underlying the apoptosis of AT2 cells is still unclear.

COPD pathogenesis is likely associated with apoptosis^79^ (Fig. 2). Inhaled oxidant from cigarette smoking and increased amount of reactive oxygen species (ROS) produced by numerous inflammatory cells in the airways of COPD patients, leads to oxidative DNA damage of host cells^80^ and consequently activates the intrinsic apoptotic cascade facilitated by an atypical immune response with the predominance of CD8+ cytotoxic cell^81^. While little is yet acknowledged about the mechanisms underlying apoptosis of AT2 cells in COPD, experiments to describe them will be fascinating and captivating.

### The role of AT2 cells in repairing processes after lung injury

The cellular and molecular mechanisms underlying how AT2 cells involved in the repair of an injured alveolar barrier is still debatable. Electron microscopy reveals that there are intermediate cell types shown in NO_2_-injured lung that display morphological characters of both AT2 and AT1 cell^82^. A potential mechanism to trigger AT2 cell activation into the repair process may be signals associated with the injury. Recent studies have proposed that the inflammatory milieu that forms after most types of alveolar injury can generate alveolar regenerative signals^83^. In injuries prompted by hyperoxia, the formation of oxidants may also signal the commencement of the repair process^84^. AT2 cells, in response to unidentified signals connected to definite injuries, can start proliferation and AT1 cell transition which may also migrate to the injured cells for reparation.

### The mechanism AT2 cells activation promote repairing processes after lung injury

**Fig. 4:**
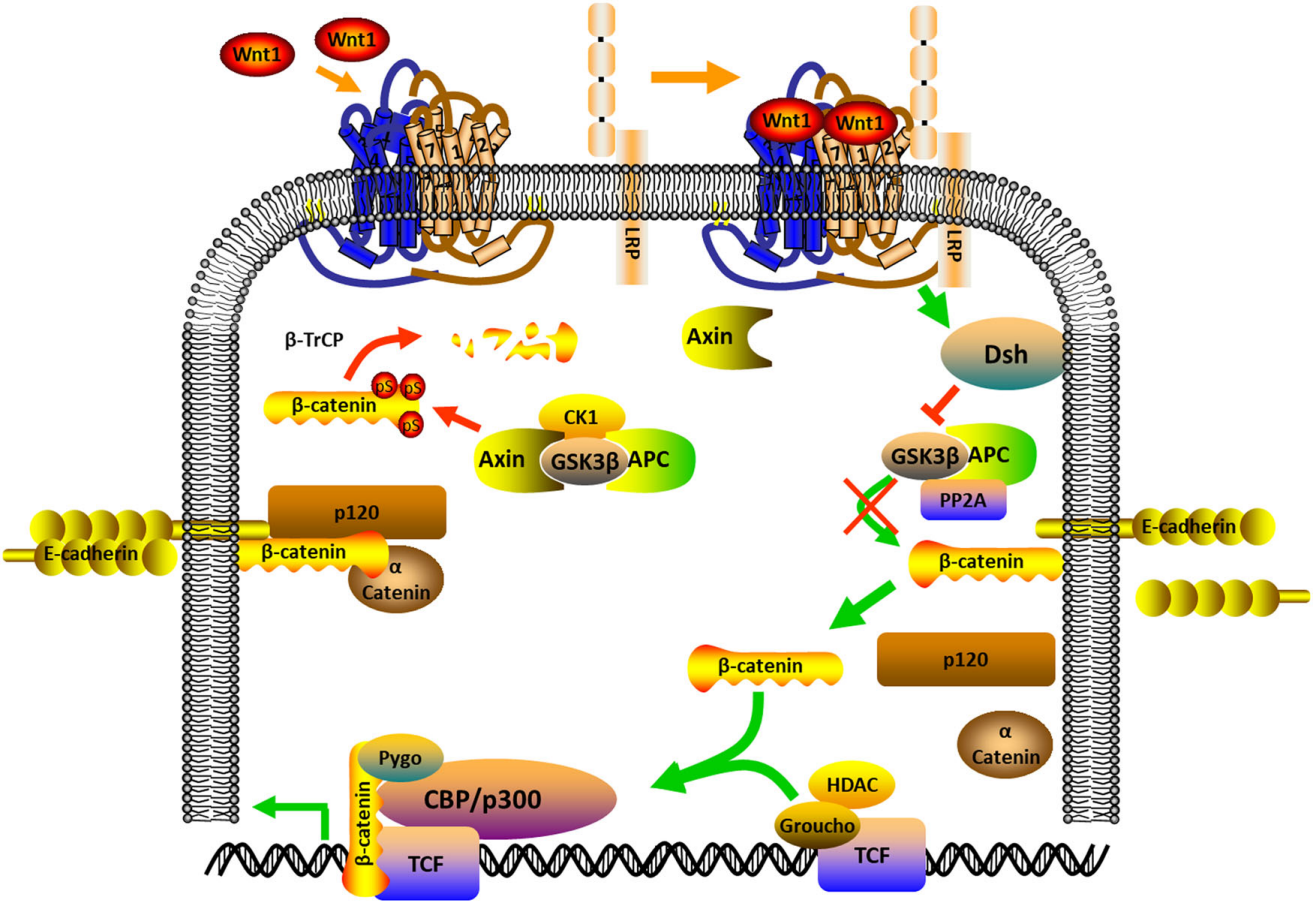
**Wnt signaling pathway**

Little is known concerning the molecules that control the AT2 cell activation that results to alveolar repair. The assumption of transcriptional programs engaged in embryonic lung development, such as those under the regulation of the FGF and Wnt pathways (Fig. 4) may be triggered after injury and may assist in repair^85^. However, even though FGF and Wnt signaling appear to be associated with alveolar repair numerous transcription factors (Id2, Erm, Gata6, and Elf5) that are involved in alveolar growth have not been revealed to have high expression in AT2 cells after Pseudomonas aeruginosa-induced injury^86^. Hence, there may be some associations of the repair development that does not absolutely summarize the developmental process in embryogenesis. Some growth factors seem to be capable to regulate definite aspects of the progenitor properties of AT2 cells. EGF and HGF can also trigger cultured AT2 cells to proliferate^87^. The intratracheal injection of HGF or KGF stimulates AT2 cell proliferation in the lung. However, TGFβ expression in the bleomycin-injured lung indicates that it shows a negative regulatory function in the proliferation of AT2 cells during early repair phase^88^. In culture, TGFβ inhibits AT2 cell proliferation but stimulates the transformation of cultured AT2 cells into AT1 cells^89^, however, BMP4, another member of the TGF superfamily, antagonizes this differentiation. The Wnt/β-catenin signaling pathway^90^ shows a significant function in the differentiation of lung epithelial cells during growth. However, following the reduction of Notch endorsed the production of SPC-positive cells and the differentiation of alveolar epithelial cells, facilitating normal repair^91^. Hence, in LNEP, Hif1a deletion or upregulation of Wnt/β-catenin activity stimulated differentiation into the normal SPC-positive AT2 cell and enhanced repair^92^. This investigation highlights the detail that not entirely repair of cells are equal and reveals instances of signals that fine-tune tissue repair, such as Hif1a and Wnt/β-catenin signaling. Hence, consideration of the equilibrium between good and bad repair is very important. More understanding of the mechanism of the signals that can determine normal versus abnormal lung repair is essential. The immune signaling functions of the Notch in the cell occur majorly in the inflammatory cells^93^. Therefore, it is promising that sustaining Notch signaling might reduce effective lung repair by extending inflammation, as well as by regulating progenitor identity, while this remains to be exploited.

### Potential therapy of lung disease by utilizing the type 2 progenitor cells

The fibrotic response could be prevented by the use of pharmacological inhibition of apoptosis. It was discovered that bleomycin-induced accumulation of lung collagens could be blocked by daily intraperitoneal injections of N-benzyl carboxy-Val-Ala-Asp-fluoro methyl ketone (ZVADfmk), a broad spectrum inhibitor of caspases (cysteine proteases) essential for the induction of programmed cell death. Shortly another investigator confirmed the blockade by using the same caspase inhibitor (ZVADfmk) administered by aerosol to mice^94^. Another approach to interrupt AT2 apoptosis showed active in blocking bleomycin-induced pulmonary fibrosis. The forced expression of p21, mainly in lung epithelial cells, utilized both antiapoptotic and antifibrotic effects.

In spite of uncertainties about their safety and the best administration route to ameliorate their engraftment, use of stem cells in animal models has been validated to mitigate injury and fibrosis in lungs confronted with endotoxin^95^. The explicit mechanisms by which stem cells accomplish their roles in tissue repair are still under study, the inhibition of pro-inflammatory cytokines and release of several growth factors seem to be involved. The utilization of exogenous growth factors is an alternative therapy used to induce the proliferation of endogenous stem cells. EGF, KGF, and HGF have been utilized. They are mitogens in AT2 cells and facilitate their maturation by increasing surfactant production synergistically. EGF had significant effects in an animal model of ALI, and inhibition had bad effects in reparation^96^.

Modulation of epithelial cell migration and anti-inflammatory cytokines are been upregulated by KGF. ATP-citrate. stearoyl CoA desaturase, lyase, fatty acid synthase, and acetyl CoA carboxylase are the major enzymes of fatty acid biosynthesis that are activated by KGF. They are controlled by two sets of enzymes primarily by two sets of transcription factors, SREBP-1c, and C/EBP alpha and delta. Transcription and proteolytic processing level controlled by SREBP-1c, LXR agonist TO901317SREBP-1c can also activate SREBP-1c^97^. Regulation at the level of phospholipid synthesis in vitro and in vivo is less clear. AT2 cells use the fatty acids for phospholipid synthesis. Phospholipid synthesis is a part that requires further study which can enhance the therapeutic effect of AT2 cells.

Vascular endothelial growth factor (VEGF) could also have a therapeutic effect by its capability to repair injured endothelium, consequently facilitating in clearance of lung edema, but animal models have displayed unsatisfactory results. Nevertheless, no study has explicitly investigated the effects of steroids throughout lung repair MMPs are other targets to encourage repair^98^. Though, the route of administration could be appropriate, as a recent trial has established that patients with ALI treated with inhaled salbutamol displayed no important improvement. Furthermore, blockade of MMP-8 has displayed favorable results in experimental models of lung injury, with reduced lung fibrosis after bleomycin administration^99^. Conversely, no clinical investigation on reducing this protease has yet been recommended.

## Conclusions

AT2 progenitor cells, growth factors, or drugs that promote matrix remodeling could be another possibility to advance the therapy of patients with lung injury. The mechanisms that can cause tissue disorder in the early phase likewise contribute to its repair, later on, inflammation and matrix renovation being model illustrations. Hence, therapies that disorder these pathways, such as MMP inhibition, may have a prophylactic value, but their application at a later phase could be detrimental. Hence, understanding of the intermediaries involved in tissue repair could lead to new therapeutic strategies being applied after the initial insult has been measured.

The accumulating knowledge regarding AT2 progenitor cells would be improved by further studies of the following directions, how is phospholipid production structured for surfactant production and association with the hydrophobic surfactant proteins, what controls the fate of dividing AT2 cells and what regulates their proliferation in the normal lung, are all AT2 cells equipotent in terms of surfactant production, fluid transport, re-epithelialization, and immune responses. It is also interesting to investigate the effects of ROS (Hydrogen peroxides, nitric oxide, and hydroxide) on induced DNA damage and repair through the differentiation of AT2 cells progenitor cells. The significance of mitochondrial complexes I and III, NADPH oxidase isoform NOX4 during AT2 cell differentiation and mechanisms underlying the processes will be fascinating to study.
